# P-2173. Respiratory Virus Detections among Asymptomatic Students and Staff Members in a Large Public School District in Kansas City, Missouri, 2023-2025

**DOI:** 10.1093/ofid/ofaf695.2336

**Published:** 2026-01-11

**Authors:** Brian R Lee, Jennifer E Schuster, Brittney Fritschmann, Olivia Almendares, Hannah L Kirking, Nibha Sagar, Dithi Banerjee, Anjana Sasidharan, Rangaraj Selvarangan, Jennifer Goldman

**Affiliations:** Children's Mercy Kansas City, Kansas City, Missouri; Children's Mercy Kansas City, Kansas City, Missouri; Children's Mercy Hospital, Kansas City, Missouri; Centers for Disease Control and Prevention, Atlanta, Georgia; Coronavirus and Other Respiratory Viruses Division, National Center for Immunization and Respiratory Diseases, CDC, Atlanta, GA; Children's Mercy hospital, Kansas City, Missouri; Children's Mercy Hospital, Kansas City, Missouri; Childrens Mercy Hospital, Missouri, Kansas; Children’s Mercy Hospital, Kansas City, Missouri; Children's Mercy Hospital, Kansas City, Missouri

## Abstract

**Background:**

While the epidemiology of acute respiratory illness (ARI) among those seeking medical care is well studied, less is known about ARI in non-medical settings (e.g., schools), especially in individuals not exhibiting ARI symptoms. We examined respiratory virus detections among asymptomatic students and staff in a public school district.

**Table 1:**
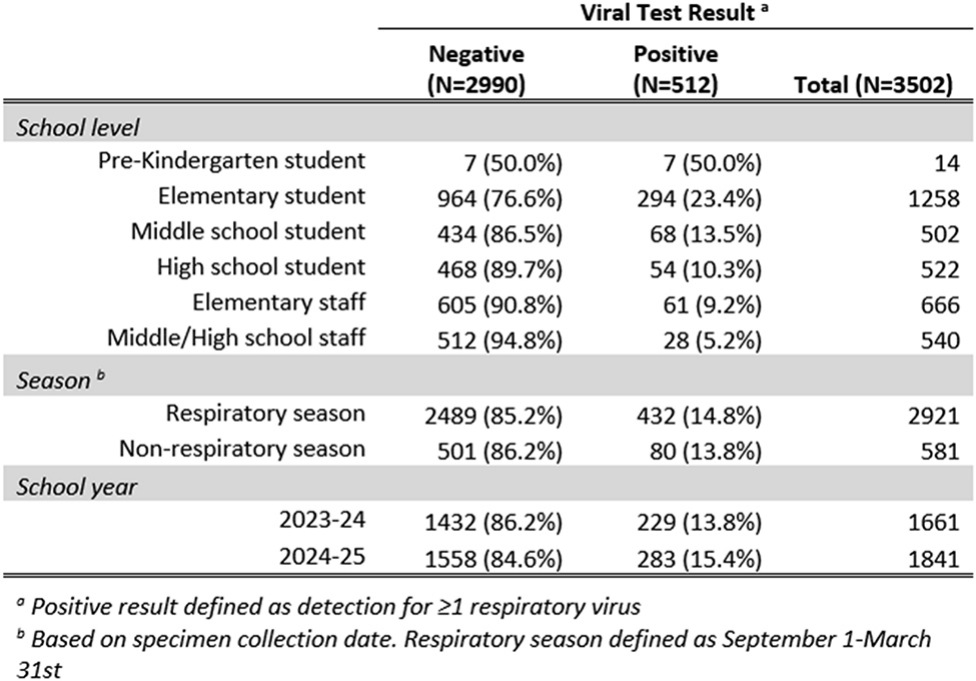
Frequency of Respiratory Viral Detection Among Respiratory Surveillance Swabs Collected from Students/Staff Reporting No Recent Acute Respiratory Illness Symptoms

**Figure 1:**
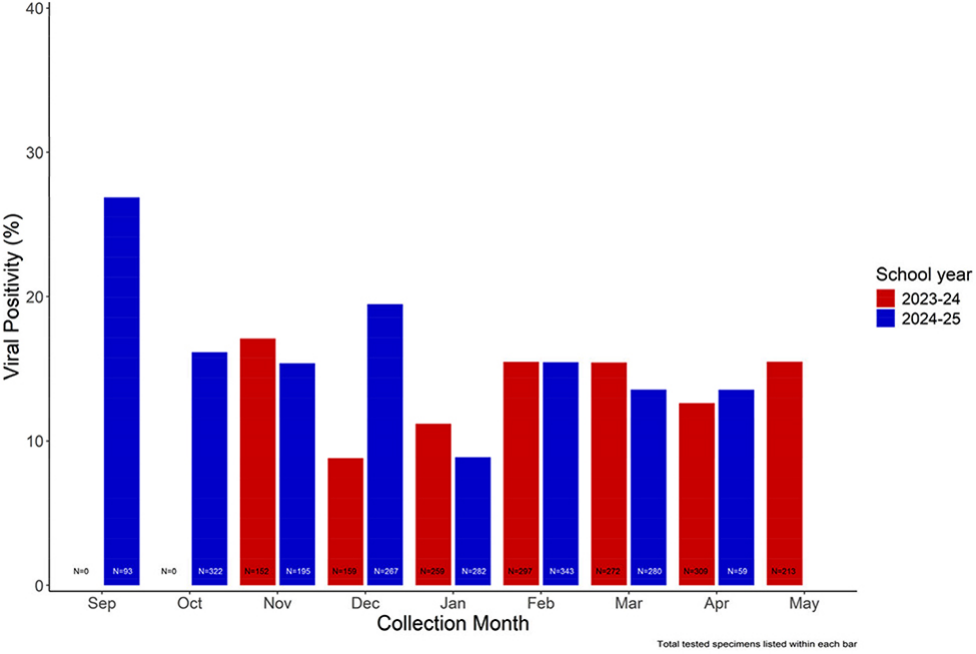
Viral Positivity Among Respiratory Surveillance Swabs Collected from Students/Staff Reporting No Recent Acute Respiratory Illness Symptoms, by Collection Month and School Year Note: Viral positivity defined as detection of ≥1 respiratory virus from a respiratory surveillance swab. Testing for the 2023-24 school year began in November 2023. Testing for the 2024-25 school year is ongoing.

**Methods:**

Knowledge of Infectious Diseases in Schools (School KIDS) is a prospective respiratory virus surveillance program in a preK-12^th^ grade public school district in Kansas City, MO. From 2023—2025, student/staff participants self-collected monthly anterior nares swabs. Specimens were tested by multiplex PCR for adenovirus, human metapneumovirus, influenza, parainfluenza, respiratory syncytial virus, rhinovirus/enterovirus (RV/EV), seasonal coronaviruses (sCoV), and SARS-CoV-2. Prior to specimen collection, participants were asked about ARI symptoms (cough, fever, congestion, runny nose, shortness of breath, sore throat, and wheezing) in the past 7 days. Specimens from participants with no ARI symptoms (i.e., asymptomatic) were included in the analysis. Differences between positive (detecting ≥1 virus) and negative specimens were assessed. We used multilevel logistic models to compare odds of viral detection adjusting for school-level and season.

**Figure 2:**
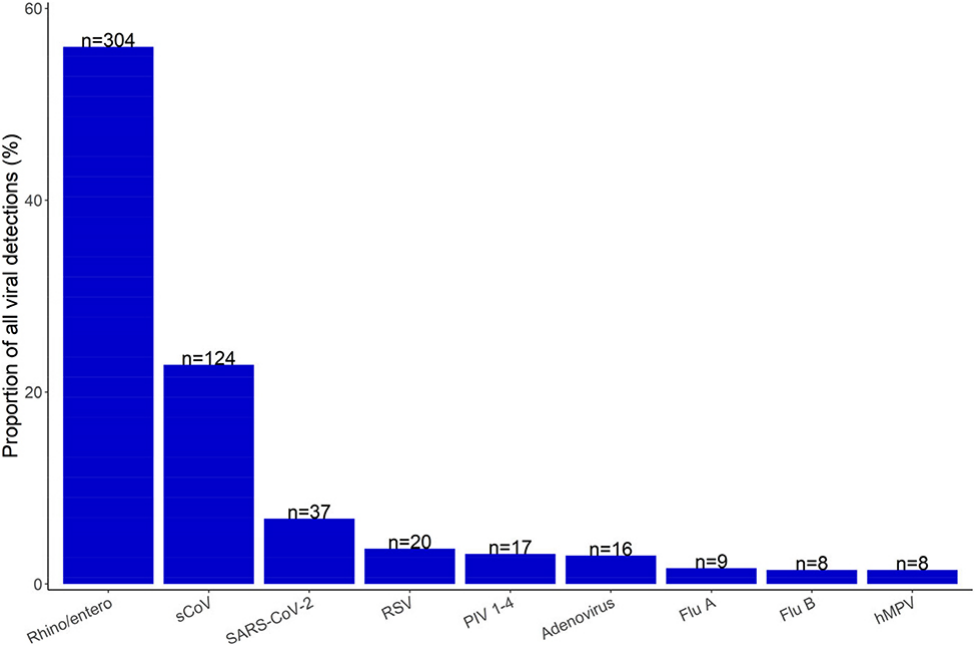
Proportion of Virus Detections in Respiratory Swabs Collected from Students/Staff Reporting No Recent Acute Respiratory Illness Symptoms by Specific Virus Note: the numbers above each bar indicate the frequency of positive detections. Of the 512 positive swabs, 27 had more than one virus detected. Therefore, total detected viruses were 543.

**Table 2:**
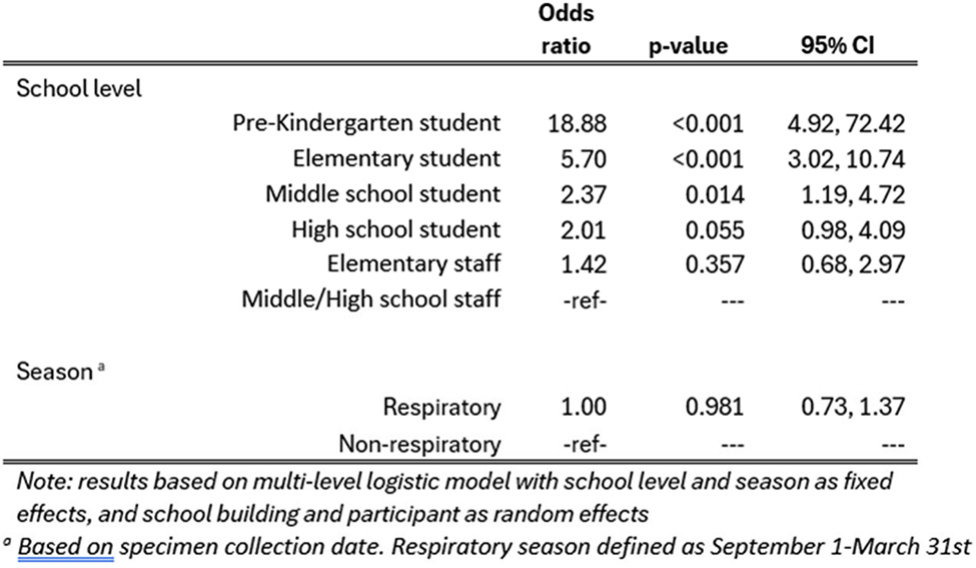
Adjusted Odds of Detecting ≥1 Respiratory Virus Among Respiratory Surveillance Swabs Collected from Students/Staff Reporting No Recent Acute Respiratory Illness Symptoms

**Results:**

Of 5398 specimens collected from 2023—2025, 3502 (65%) were from asymptomatic participants. Viral positivity among these specimens was 15% (n=512/3502). Viral positivity varied by school level (Table 1) but overall showed little seasonal variation (Figure 1). Most commonly detected viruses were RV/EV (56%), sCoV (23%) and SARS-CoV-2 (7%) (Figure 2). Compared with staff from middle/high-schools, increased odds of viral positivity were observed for preK (OR: 18.9 [4.9, 72.4]), elementary (OR: 5.7 [3.0, 10.7]), and middle-school students (OR: 2.4 [1.2, 4.7]) (Table 2).

**Conclusion:**

Viral detections in asymptomatic students and staff were frequent (15%), with relatively consistent positivity (for any virus) throughout the school year. Public health strategies to mitigate respiratory viral transmission including cough/hand hygiene and staying home while sick, among others, may reduce both symptomatic and asymptomatic transmission

**Disclosures:**

Brian R. Lee, PhD, MPH, Merck: Grant/Research Support Rangaraj Selvarangan, PhD, Altona: Grant/Research Support|Biomerieux: Advisor/Consultant|Biomerieux: Grant/Research Support|Biomerieux: Honoraria|Cepheid: Grant/Research Support|Hologic: Grant/Research Support|Hologic: Honoraria|Meridian: Grant/Research Support|Qiagen: Grant/Research Support

